# Employees and the National Insurance Act

**Published:** 1912-08-31

**Authors:** 


### NATIONAL INSURANCE ACT DEPARTMENT.

### EMPLOYEES AND THE NATIONAL INSURANCE ACT.

### XXVI.—Transfers.

There are several ways in which the question of transfer arises under the Act. In the first place there may be transfer of membership from one society to another. Then there may be transfer from membership of an approved society to a similar foreign or colonial society. Again, members of approved societies may elect to discontinue their connection with their societies and become deposit contributors. Deposit contributors may also become members of approved societies. Transfers will further have to be recorded in the books of the approved society for those members who remove from England to Wales, or from Scotland to England, or from any one country of the Union to any other. This question of transfer also involves questions of finance, and special attention had to be directed to it in the Act.

#### Transfer from one Society to Another.

Transfer from one society to another may occur in either of two ways. It may be voluntary on the part of the insured person or it may be occasioned by his expulsion from membership of his society. Expulsion from a society can only be effected in conformity with the rules of the society—on such grounds, for instance, as that the member has committed an offence against the rules, or that he has removed to a place outside the district in which the society conducts its operations. It may here be remarked that in the case of societies formed by employers for the benefit of their staffs the power of expulsion is not allowed to be exercised when a member is discharged from or leaves the service of his employer if he is unable to obtain admission to another society on account of the state of his health. When a member leaves his society it will be necessary for his "transfer value" to be ascertained according to tables to be prepared by the Insurance Commissioners. The "transfer value" will be somewhat of the nature of the "reserve values" of which we have spoken in recent issues; in fact, at the commencement of the Act the "transfer value" and the "reserve value" of a member are identical. On entry into insurance each person who joins an approved society is to be credited with a reserve value appropriate to his age; if above age sixteen, and for some time to come those who are below middle age, taken as a whole, will contribute more to the funds of the society, from the weekly contributions and the interest earned on the reserve values, than the amount that will have to be paid them in benefits. The balance will have to be added to the reserve and invested at interest. In course of time the cost of the benefits to be provided by the funds of the society will be more each year than the contributions and interest received on account of these members; and then the time will come for drawing on the reserves.

In similar fashion those who are beyond middle age on entry into insurance will, as a class, draw more in benefits than they add to the funds of the society by their contributions and the interest earned on their reserves. Thus it happens that in the case of young lives the reserve values held by the societies will increase from the time of entry to a maximum at about age fifty, and will thereafter diminish until at age seventy the only value remaining in the fund will be such as to ensure the continuation of medical benefit for the remainder of life. This is, of course, only a rough idea of the progress of the insurance funds that will have to be accumulated by the societies, but it is sufficient to show that there will be in the funds of each society a certain sum on account of each member depending upon his age at entry and the duration of his membership. The insurance could not be continued without this accumulated fund, and, consequently, when a member transfers from one society to another, it is necessary that the sum should be transferred with him. Hence the description "transfer value."

No society can view with unconcern the secession of its members, particularly if those seceding are in good health, thus leaving in the society more than its due proportion of unhealthy lives. From the point of view of the society which is losing a member the desire will be for the transfer value to be assessed as small as possible, because it will not wish its funds to be depleted. On the other hand, the society to which transfer is being made will wish the transfer value to be as large as possible. Thus, as transfer is to be allowed under the Act, it is necessary that there should be a standard table of reference applicable to all societies, and great care will have to be exercised in its calculation to ensure equitable treatment to all parties. It is open to any society to reject an application for membership on any ground other than age, and, unless the transfer values are framed on an adequate scale, it is to be feared that a member of an approved society wishing to transfer will find difficulty in obtaining admission to any other society.

If a member is expelled, the society expelling him is bound in every case to relinquish his transfer value, but if the member discontinues his membership voluntarily the society has the right of withholding its consent, and if it can prove that its consent was not unreasonably withheld, it will not have to part with the transfer value, which will in that event fall into the general fund of the society.

These arrangements as to transfer from one society to another apply equally to transfers from one branch to another branch of the same society.

#### Transfers to Foreign and Colonial Societies.

In normal cases if an insured person ceases to be permanently resident in the United Kingdom he ceases to be insured, but if the insured person is a member of an approved society and upon permanent removal abroad becomes a member of a society or institution of a similar character, which is approved by the Insurance Commissioners, or of a foreign or Colonial branch of an approved society, his transfer value will be paid to that. This provision as to payment of transfer values is conditional upon the society giving reciprocal rights to any of its members who become resident in the United Kingdom. If the insured person is a deposit contributor the amount standing to his credit in the Post Office Fund may similarly be transferred. When an arrangement has been entered into between the British Government and any foreign or Colonial Government for the transfer of insured persons on reciprocal terms, the payment of the transfer value will be allowed in all cases without special regard to the merits of the particular society or institution.

From Approved Society to Deposit Insurance.

As it is so manifestly to the advantage of an insured person to become a member of an approved society rather than a deposit contributor, it is hardly likely that anyone who has become a member of a society will voluntarily relinquish membership to become a deposit contributor. It may be, however, that upon expulsion from his society an insured person is unable to gain admittance to another society. Whether such transfer is voluntary or due to expulsion from his society the insured person will lose the right to the reserve value that was credited to his society on entry. Only the excess, if any, of the transfer value over the reserve value will be credited to his account in the Post Office Fund, and the reserve value will be cancelled.

Deposit Contributors to Approved Societies.

The opposite case of the transfer of deposit contributors to approved societies will be much more common. In this case the reserves that would have been credited to the society if the insured person had been a member on entry into insurance will be credited to the society, less any sums by which such reserve would have been reduced by the process of writing down. The amount standing to his credit in the Post Office Fund will then be made use of, so far as it will go to make up the reserve value to the transfer value as at the date of entry into the society. If the amount standing to his credit is insufficient for the purpose, the member will be treated as in arrear to the extent of the deficiency, whereas if the amount is more than sufficient only the sum required will be transferred and the balance will be devoted to the general purposes of the Post Office Fund. There is, therefore, some consolation for those who become deposit contributors in the fact that they need not remain in that category always if they can find a society willing to admit them to membership, and upon entry into the society they are put into virtually as good a position as though they had originally joined.

Transfers by Removal.

Transfers from one society to another and from approved societies to deposit insurance and vice versa are not likely to be of any serious consequence for some little time to come, though there is some doubt as to what is to constitute membership of a society at the outset, and if it is necessary for the process of transfer to be gone through in October next when the first insurance card is not returned to the same society as the one through which it was issued. Transfers by removal, while not affecting the membership, have already occasioned an amount of work, and in course of time they may occasion some trouble and inconvenience. This is particularly in evidence in the case of societies transacting business in all four countries of the United Kingdom. It was not the intention of the original draft of the Act to require separate funds for the separate countries, but an amendment to this effect was pressed and eventually incorporated into the Act. As an outcome it is necessary that every insured person should be placed on the fund of his society for the country in which he resides. In the border counties between England and Wales and between England and Scotland migration is continuous and considerable in extent, so that the matter has already occasioned some anxiety to those who have to record these constant changes. It is too early to estimate the precise effect upon the benefit funds of the societies, though it has been argued that as the flow of migration from Scotland to England is that of younger persons than the corresponding migration from England to Scotland the Scotch funds will have to bear the heavy charges for sickness in old age, while the English funds will reap the benefit of the payment of contributors in earlier life. This is, of course, merely a matter for argument and cannot be supported by precise figures. It shows, however, that the additional administrative organisation set up by the institution of separate funds for the four countries of the Union will not necessarily retain for each country its own surplus.

### Answers to Correspondents.

### Provision of Surgical Appliances.

X. Y., Liverpool, writes: "I see surgical appliances are supplied under the Insurance Act. Are artificial limbs and false teeth included? If so, when can they be obtained?"—The provision of surgical appliances is part of the medical benefit conferred by the Act, and it is expressly stated (Section 8 (1) (a) that the appliances will be such as may be prescribed by regulations to be made by the Insurance Commissioners. These regulations have not yet been published, and we cannot, therefore, reply definitely to the questions, but we may say frankly that we very much doubt if either of the appliances mentioned will be included in the list. In any case the prescribed appliances cannot be obtained until after January 15 next, when medical benefit begins to accrue.

### Seamstresses and National Insurance.

L. E.—A seamstress in regular work for an employer will be compulsorily insured under the Act, subject to the usual provisions as to the rate of remuneration not exceeding £160 per annum. If working on her own account she may become a voluntary contributor. In either case she will be entitled to the State benefit of one quarter the amount paid in benefits and in their administration. The State grant is not paid in the form of 2d. a week in addition to the weekly contribution.

